# An Arrhythmia Classification Model Based on Vision Transformer with Deformable Attention

**DOI:** 10.3390/mi14061155

**Published:** 2023-05-30

**Authors:** Yanfang Dong, Miao Zhang, Lishen Qiu, Lirong Wang, Yong Yu

**Affiliations:** 1 School of Biomedical Engineering, Division of Life Sciences and Medicine, University of Science and Technology of China, Hefei 230026, China; 2 Suzhou Institute of Biomedical Engineering and Technology, China Academy of Sciences, Suzhou 215163, China; 3 School of Electronics and Information Technology, Soochow University, Suzhou 215031, China

**Keywords:** arrhythmia, deep learning, ECG signal, deformable attention transformer, depthwise separable convolution

## Abstract

The electrocardiogram (ECG) is a highly effective non-invasive tool for monitoring heart activity and diagnosing cardiovascular diseases (CVDs). Automatic detection of arrhythmia based on ECG plays a critical role in the early prevention and diagnosis of CVDs. In recent years, numerous studies have focused on using deep learning methods to address arrhythmia classification problems. However, the transformer-based neural network in current research still has a limited performance in detecting arrhythmias for the multi-lead ECG. In this study, we propose an end-to-end multi-label arrhythmia classification model for the 12-lead ECG with varied-length recordings. Our model, called CNN-DVIT, is based on a combination of convolutional neural networks (CNNs) with depthwise separable convolution, and a vision transformer structure with deformable attention. Specifically, we introduce the spatial pyramid pooling layer to accept varied-length ECG signals. Experimental results show that our model achieved an F1 score of 82.9% in CPSC-2018. Notably, our CNN-DVIT outperforms the latest transformer-based ECG classification algorithms. Furthermore, ablation experiments reveal that the deformable multi-head attention and depthwise separable convolution are both efficient in extracting features from multi-lead ECG signals for diagnosis. The CNN-DVIT achieved good performance for the automatic arrhythmia detection of ECG signals. This indicates that our research can assist doctors in clinical ECG analysis, providing important support for the diagnosis of arrhythmia and contributing to the development of computer-aided diagnosis technology.

## 1. Introduction

Cardiovascular disease (CVD) is the leading cause of death, accounting for over 32% of all deaths worldwide [1]. Cardiac arrhythmia (CA) serves as a warning signal for cardiovascular disease and enables clinicians to provide timely interventions through early diagnosis. Electrocardiogram (ECG) is an effective non-invasive tool for monitoring heart activity and diagnosing CA [2]. Precisely detecting arrhythmia has become a significant focus for biomedical researchers. However, accurately recognizing these complex CA-associated ECG rhythms requires considerable clinical experience and expertise. Manual detection of arrhythmia consumes considerable time for expert clinicians and cardiologists and it can be a task prone to errors even for these human experts. In fact, research shows that cardiologists or diagnosing doctors sometimes misjudge the type of arrhythmia [3,4]. The introduction of computer-aided diagnosis has aimed to enhance accurate ECG interpretation and reduce costs. Consequently, it has become increasingly essential to automatically detect arrhythmia using ECG signals, as this can assist clinical diagnosis during ECG analysis [5].

As digital ECG data become more widely available and deep learning algorithms continue to advance [6], an increasing number of researchers are turning to deep learning approaches for automated arrhythmia detection. These studies have demonstrated that automated feature extraction, as opposed to manual ECG morphological feature extraction, leads to more accurate prediction results when using deep-learning-based methods [7,8,9]. In the automatic ECG analysis algorithms based on deep learning, convolutional neural networks (CNNs), which can achieve data feature extraction through local receptive fields, weight sharing, downsampling, and other methods [10], are usually used as the backbone to extract features automatically. For instance, Kiranyaz et al. proposed an adaptive 1D CNN model that integrates feature extraction and classification into a single learning body for ECG classification. The model was trained using relatively small sets of common and patient-specific training data and achieved remarkable accuracy [11]. Rajpurkar et al. proposed a 34-layer CNN for classifying 14 types of cardiac arrhythmias. The model was trained end-to-end on a single-lead ECG signal and outperformed the average cardiologist in terms of both recall (sensitivity) and precision (positive predictive value) [12]. Acharya et al. utilized an 11-layer CNN algorithm for automated detection of normal and myocardial infarction ECG beats. Their model was able to accurately detect unknown ECG signals even in the presence of noise [13]. He et al. developed a 2D CNN for detecting atrial fibrillation (AF) episodes, achieving sensitivity, specificity, positive predictive value, and overall accuracy rates of 99.41%, 98.91%, 99.39%, and 99.23%, respectively [14]. Jun et al. proposed an effective ECG arrhythmia classification model that utilizes two-dimensional convolutional neural networks with ECG images as an input [15]. Then, in the detection of life-threatening cardiac arrhythmias, Elola et al. proposed two deep neural network (DNN) architectures to classify the rhythm into pulseless electrical activity (PEA) or pulse-generating rhythm (PR) using short ECG segments, and both architectures achieved excellent performance [16]. Dubatovka et al. explored deep neural networks (DNNs) for learning cardiac cycles and reliably detecting AF from single-lead electrocardiogram (ECG) signals with a superior performance [17]. Krasteva et al. reported the optimal hyperparameters of an end-to-end fully convolutional DNN architecture for detecting shockable and nonshockable rhythm using single-lead raw ECG signals with life-threatening arrhythmias [18]. Jekova et al. optimized the architecture of a computationally efficient end-to-end CNN models for ECG rhythm analysis during cardiopulmonary resuscitation [19].

Although the aforementioned methods have achieved great success in the detection of ECG arrhythmia, it is widely acknowledged that temporal information plays an important role in tackling even more complex arrhythmia detection problems [20]. As a time series of data, ECG signals inherently contain temporal dependencies within their waveform. Recurrent neural networks (RNNs) can capture temporal dependencies in sequential data more efficiently compared to CNNs [8]. For example, Wang et al. proposed a global and updatable classification scheme called the global RNN (GRNN) in which RNN was used for automatic feature learning and classification based on the morphological and temporal information of ECG [21]. Recognizing the unique characteristics of ECG signals, Chen et al. achieved excellent performance in arrhythmia classification by fusing the CNN and RNN models [22].

In recent years, the transformer has become a popular deep learning model alongside CNNs and RNNs. It utilizes an attention mechanism to capture temporal features and context vectors and was originally developed for natural language processing (NLP) tasks [23,24,25,26]. However, it has also demonstrated superior performance on various vision tasks [27,28], including image classification. One representative work is the vision transformer (ViT) [29], which processes images as sequences of patches using a standard transformer encoder used in NLP. Compared to CNNs, transformer-based models have larger receptive fields and excel at modeling long-range dependencies, resulting in better performance on many image classification data sets [30,31]. As ECG signals exhibit temporal dependencies in their waveforms, the ViT’s mechanism can be applied to arrhythmia classification tasks. Yan et al. proposed a heartbeat classification model based on a transformer that utilized the encoder part to process segmented single-lead ECG signals [32]. Natarajan et al. developed a wide and deep transformer neural network that combined handcrafted ECG features determined by a random forest model with discriminative feature representations automatically learned from a transformer neural network to classify each 12-lead ECG sequence into 27 cardiac abnormality classes [33]. Che et al. embedded a transformer network in a CNN to capture the temporal information of ECG signals for arrhythmia classification while introducing a new link constraint to the loss function to enhance the classification ability of the embedding vector [34]. These studies demonstrate the effectiveness of utilizing the transformer network structure for solving arrhythmia classification problems. However, due to the interference on the ECG waveform morphology of different diseases and the complex relationship between them, existing transformer approaches still have several limitations. 

In summary, the existing automatic ECG analysis algorithms that solely used CNN cannot fully exploit the temporal features of ECG signals. Compared to CNN, RNNs can more efficiently capture temporal dependencies in sequential data, and the models fusing CNN and RNN have demonstrated excellent performance in arrhythmia classification. However, these models typically use isolated heartbeat signals as input, which results in a failure to leverage inter-heartbeat dependencies and requires the explicit segmentation of heartbeats. The segmentation of heartbeats usually necessitates the use of QRS detection algorithms such as Wavedet [35] and Pan–Tompkins [36]. For real-world scenarios, this means that an additional preprocessing step is required. As mentioned in the previous paragraph, the transformer architecture can capture temporal features through an attention mechanism and research has demonstrated the effectiveness in solving arrhythmia classification problems. However, the attention mechanism in the current approaches based on the transformer has a wide receptive field that can include irrelevant information outside the region of interest, affecting the amplitude and local statistical information from ECG signals. Moreover, the majority of existing methods take equal-length ECG signal segments as input. To address these issues, we propose the CNN-DVIT model, an end-to-end multi-label classification model that combines CNN with depthwise separable convolution and a vision transformer structure with deformable attention. 

The major contributions of our model are as follows: Firstly, our approach involves replacing the multi-head self-attention mechanism in the original vision transformer model with a more effective deformable self-attention module, which enables the self-attention module to focus on relevant regions and capture more informative features. Secondly, we introduce the spatial pyramid pooling layer to accept variable-length 12-lead ECG signals as input, which eliminates the explicit segmentation of heartbeats beforehand and can make use of inter-heartbeat dependencies to improve the classification performance. Thirdly, to further mine the information of every lead for multi-lead ECG signals, the depthwise separable convolutions replace the conventional convolution in the CNN backbone.

## 2. Methods

In this section, we will initially provide an overview of the model’s overall structure, and then proceed to introduce the specific structure of each individual component.

### 2.1. Model Architecture

**Figure 1:**
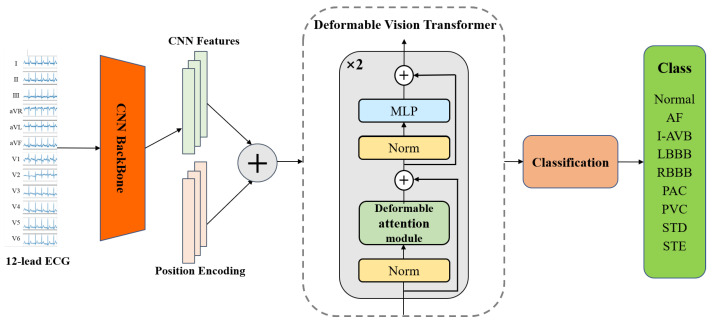
Overview of the proposed model.

Our proposed model, illustrated in Figure 1, is able to take continuous 12-lead ECG signals as input and output the arrhythmia diagnosis result in an end-to-end manner. Concretely, the model is composed of three main components: (1) a CNN-based backbone for feature extraction from each lead; (2) a deformable attention transformer encoder module to combine the CNN-extracted features and the positional encoding; and (3) the classification layer to obtain the probability that each patient may have for each type of heart disease. The first part, the CNN-based backbone, is based on the original Inception module with residual connections, in which the depthwise separable convolutions replace the conventional convolutions. We attempt to extract details of waveform variation from every lead of ECG by using depthwise separable convolutions that operate independently on each lead. Following the CNN-based backbone, the extracted features combined with positional encoding pass through the deformable attention transformer encoder module. The output from the deformable transformer encoder is then fed into the classification layer, which generates the predicted probability distribution over the nine classes.

### 2.2. CNN-Based Backbone

**Figure 2:**
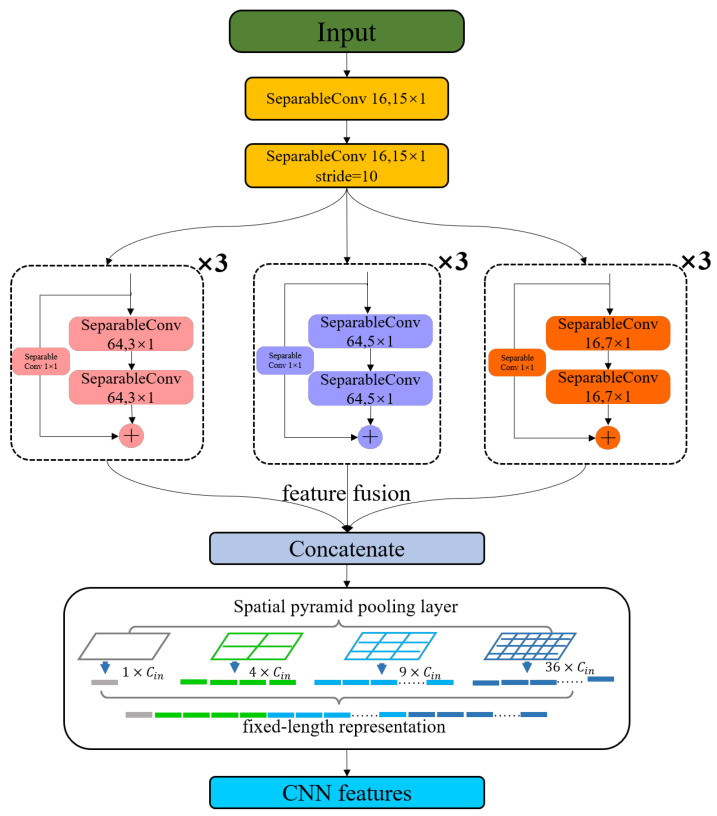
This is the structure of CNN Backbone.

CNNs can extract data features using local receptive fields, weight sharing, downsampling, and other methods [10], which are commonly used as the backbone for feature extraction due to their ability to capture local features and translation invariance [37]. The depthwise separable convolutions were utilized in the Xception architecture developed by Google and demonstrated enhanced performance in image classification [38]. A depthwise separable convolution consists of a depthwise convolution and a pointwise convolution. The depthwise convolution is a spatial convolution performed independently over each channel of an input; and then the pointwise convolution is a 1 × 1 convolution projecting the channel output by the depthwise convolution onto a new channel space. For multi-lead ECG signals, the spatial convolutions are applied to each lead, and then the feature map of every channel is projected onto a new space, enabling ECG details to be extracted from every lead. Actually, our CNN-based backbone is built on the original Inception module with residual connections, where conventional convolutions are replaced by depthwise separable convolutions. Specific configuration details are shown in Figure 2. In the early stage of this part, we use a large convolution kernel with a size of 15, increasing the receptive field of the convolution network and facilitating the detection of large-scale waveforms. In the middle, we utilize the residual network structure and Inception structure. To learn information from different scales, we employ three different scales of convolution kernels. At the end of the CNN-based backbone, we introduce the spatial pyramid pooling layer to convert the different dimensions of the final output feature map into a fixed-dimensional CNN feature without considering the length of input signals [39]. As depicted in Figure 2, we divide the output feature graph from each lead in the first part into 36 blocks, 9 blocks, 4 blocks, and 1 block, and then compute the maximum pooling for each block individually. Therefore, the model can accommodate arbitrary-length ECG signals.

### 2.3. Deformable Vision Transformer

The deformable vision transformer [40] was developed based on a deformable self-attention module, where the positions of key and value pairs in self-attention are selected in a data-dependent way. In the ECG signal classification problem, this flexible scheme enables the self-attention module to focus on relevant regions and capture more informative features to accurately identify the distinct characteristic wave types and recognize different arrhythmia categories. The structure is shown in the deformable vision transformer module in Figure 1. The deformable vision transformer network in our model contains two identical layer stacks and each layer has two sub-layers. The first sub-layer is the deformable attention module; the second is the multi-layer perceptron (MLP) block which adopts two linear transformations and a GELU activation. In addition, LayerNorm (LN) is applied before every block, and residual connections after every block [41,42].

#### Deformable Attention Module

**Figure 3:**
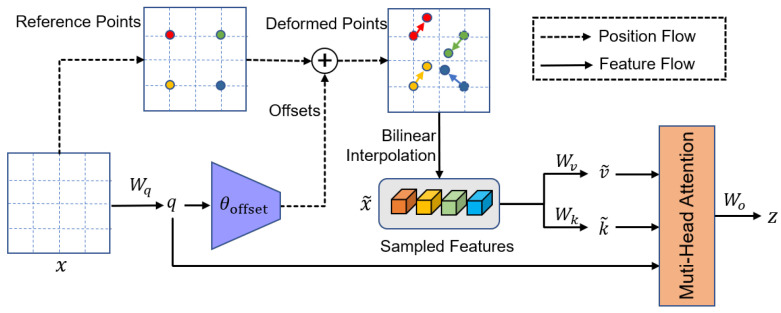
This is the structure of deformable attention module.

The ability to flexibly model relevant features is a crucial aspect of our proposed model. This crucial characteristic is implemented by the deformable attention module described in the Ref. [40], and the structure is shown as Figure 3. Specifically, the deformable attention can be viewed as a spatial adaptive mechanism to effectively model the relationship between tokens under the guidance of important regions in the feature maps. These regions are computed from sets of deformed points which are learned from the queries by an offset network. After obtaining the regions, a bilinear interpolation method is applied to sample features from the feature maps, and then the sampled features are input into the key and value projections to obtain the deformed keys and values. Finally, standard multi-head attention is applied to attend queries to the sampled keys and aggregate features from the deformed values. This can be better understood mathematically using a set of theoretical notations, as follows.

We take a feature map $x\in {\mathbb{R}}^{H\times W\times C}$ as the input, and then generate a uniform grid of reference points $p\in {\mathbb{R}}^{H/r\times W/r\times 2}$, where r is the factor by which the grid size is downsampled from the input feature map size. To obtain the offset for each reference point, the feature maps are projected linearly to obtain the query tokens q, and then fed into a lightweight sub-network offset network to generate the offsets ∆p of the reference points p. Specifically, the sub-network contains two convolution modules with a nonlinear activation. First, the input features pass through a depthwise convolution to capture local features. Then, GELU activation and a 1 × 1 convolution are adopted to calculate the offsets. After generating the offsets for the reference points, the features at the locations of deformed points are sampled by the bilinear interpolation method and then projected as keys $\tilde{k}$ and values $\tilde{v}$. Next, we perform standard multi-head attention on q, $\tilde{k}$, and $\tilde{v}$, where a multi-head attention block with M heads is formulated as:

(1) $$q=xW_{q},\;\tilde{k}=\tilde{x}W_{k},\;\tilde{v}=\tilde{x}W_{v}$$

(2) $$z^{\left(m\right)}=\sigma \left(q^{\left(m\right)}{\tilde{k}}^{(m)⊤}/\sqrt{d}\right){\tilde{v}}^{\left(m\right)},\;m=1,\ldots ,M$$

(3) $$z=\mathrm{Concat}\left(z^{\left(1\right)},\ldots ,z^{\left(M\right)}\right)W_{o}$$

where $W_{q},\;W_{k},\;W_{v},\;W_{o}\in {\mathbb{R}}^{C\times C}$ are the projection matrices, $\tilde{x}=\phi \left(x;p+∆p\right)$, $∆p=\theta_{offset}\left(q\right)$, and the sampling function $\phi \left(\cdot ;\cdot \right)$ is a bilinear interpolation as Equation (4). In Equation (2), $\sigma \left(\cdot \right)$ denotes the softmax function, and d=C/M is the dimension of each head. $z^{\left(m\right)}$ denotes the output of the m-th attention head, and $q^{\left(m\right)},{\tilde{k}}^{\left(m\right)},{\tilde{v}}^{\left(m\right)}$ represent query, the deformed key, and value embeddings, respectively.

(4) $$\phi (z;(p_{x,}p_{y}))=\sum_{\left(r_{x},r_{y}\right)}g(p_{x,}r_{x})g(p_{y,}r_{y})z[r_{y},r_{x},:]$$

where the $g\left(a,b\right)=\max\left(0,1-\left|a-b\right|\right)$ and $\left(r_{x},r_{y}\right)$ indexes all the locations on $z\in {\mathbb{R}}^{H\times W\times C}$.Finally, the features of each head are concatenated together and projected through $W_{o}$ to obtain the final output z as Equation (3). To build up a deformable vision transformer, the normalization layer and an MLP block with two linear transformations and a GELU activation are adopted after the deformable attention module [43].

### 2.4. Classification Layer

The deformable vision transformer network is connected to the classification layer for multi-classification. The classification layer consists of a full connection layer and a softmax layer, which outputs the predicted probability indicating the likelihood that each patient has each type of cardiac arrhythmia.

## 3. Experiments and Results

In this section, we will delineate the data sources, expound on the experimental design, and elaborate on the analysis of experimental results.

### 3.1. Data Description and Experiment Setup

Our paper utilized data from the 1st China Physiological Signal Challenge (CPSC-2018), consisting of 6877 12-lead ECG recordings collected from 11 hospitals [44]. The signals contained normal heart rhythms and eight types of cardiac arrhythmia, namely, Normal (N), Atrial fibrillation (AF), First-degree atrioventricular block (I-AVB), Left bundle branch block (LBBB), Right bundle branch block (RBBB), Premature atrial contraction (PAC), Premature ventricular contraction (PVC), ST-segment depression (STD), and ST-segment elevated (STE). The recordings had one, two, or three labels, with some designated as First, Second, and Third. Thus, the ECG arrhythmia detection task in our paper became a multi-label classification problem.

The model being suggested is evaluated on every data category present in the CPSC-2018 data set using a train/validation/independent-test strategy. In the ten-fold cross-validation experiment, the training data set was divided into ten parts, with nine parts used as training data and one part as validation data. After iterations of training and validation, the model with the best performance on the validation set was evaluated on unseen test data for final performance evaluation. The Adam optimizer with default parameters and a learning rate of 0.0001 was used to train the model, and the Focal Loss function was adopted as the objective loss function [45].

### 3.2. Classification Performance

#### 3.2.1. Evaluation Metrics

To measure the classification performance of our method from multiple perspectives, we introduce three performance indicators: the average *precision*, *recall* rate, and *F*1 score. The details are as follows:

(5) $$Precision=\frac{\;TP}{TP+FP}$$

(6) $$Recall=\frac{\;TP}{TP+FN}$$

(7) $$F1=\frac{\;2\times Precision\times Recall}{Precision+Recall}$$

In our multi-label classification task, for a certain class, *TP* indicates the number of correctly classified samples in this class. *FN* indicates the number of samples belonging to this class that are misclassified as in other classes. *FP* indicates the number of samples misclassified as in this class when they belong to other classes. The averages of all nine categories of the *F*1 score were used to evaluate the final performance of the model.

#### 3.2.2. Comparison with Existing Methods

To verify the performance of our proposed model, we compared our CNN-DVIT with recently proposed ECG classification methods as well as basic neural networks. Table 1 presents the results for the class-level F1 score and average F1 score of our model and six reference models. As shown in Table 1, our proposed CNN-DVIT has an average F1 score of 0.829, which is comparatively better than the other methods. Our model achieved the highest F1 score in six out of the nine ECG categories. Specifically, our approach outperformed the conventional Resnet [30] and LSTM [8] methods in all nine categories, with an average F1 score that was 0.092 and 0.165 higher than them, respectively. Moreover, the F1 scores of all categories performed better than the other methods except for PVC, where VGG-16 [10] obtained a slightly higher score. Notably, compared to the latest competitor [34], which also introduced the transformer, our CNN-DVIT outperformed in each category with an average F1 score improvement of 0.112.

**Figure 4:**
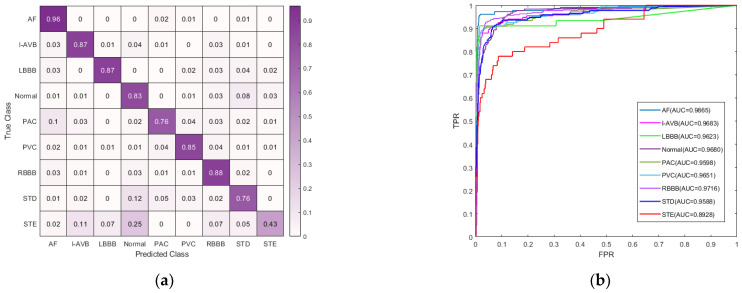
(**a**) Normalized confusion matrix of CNN-DVIT; and (**b**) the ROC curve and AUC of CNN-DVIT.

Additionally, we present the confusion matrix, receiver operating characteristic (ROC) curve, and area under the curve (AUC) for CNN-DVIT in Figure 4a,b, respectively. The confusion matrix demonstrates our model’s strong classification ability for almost all types of ECGs. However, in Figure 4a, we can observe that the PAC, STD, and STE categories exhibited relatively poor performance compared to other categories in the data set. This is attributed to a limited distribution of data within these categories. Meanwhile, the normal and STE classes demonstrated high similarity, particularly with regard to the morphology of their T waveforms. As a consequence, there was confusion and misclassification between these two classes. The ROC curve illustrates the network output at different classification criteria with FP and TP as the axis co-ordinates, effectively reflecting the classification performance of the network structure. As shown in Figure 4b, the AUC values of our CNN-DVIT model are greater than 95% for most classes, except for ST-segment elevated (STE). Therefore, it can be concluded that our model has excellent performance in classifying different cardiac diseases. These results align well with our F1 score assessment.

## 4. Discussion

In this section, we analyze the effectiveness of key components in our CNN-DVIT model through ablation experiments. We present the results for the ECG arrhythmia detection task in the CPSC-2018 data set. 

First, we assess the effectiveness of the deformable attention module. In our CNN-DVIT model, we apply the deformable multi-head attention (DMHA) module to replace the multi-head self-attention (MHSA) block of the vision transformer. We compare the classification performance of both methods, and the results are recorded in Table 2. As shown in Table 2, the average F1 score of the models that use the DMHA module is higher than those using the multi-head self-attention (MHSA) block. These experimental results indicate that the deformable multi-head attention can more effectively extract information from ECG signals and exhibit outstanding performance in ECG classification on CPSC-2018 data sets.

Next, we investigate the impact of depthwise separable convolution (DWS-CNN) in our model. We conduct an ablation experiment by comparing the DWS-CNN with conventional convolution (CNN). As shown in Table 2, models that apply the DWS-CNN have a clear advantage over those using conventional convolution. These ablation experiments demonstrate that the depthwise separable convolution (DWS-CNN), which is applied to each lead and projects the feature map of every channel to a new space, can effectively extract features of heart disease from multi-lead ECG signals for diagnosis.

## 5. Conclusions

This paper presents an end-to-end model named CNN-DVIT for arrhythmia classification of multi-lead ECG signals. The model combines a CNN backbone and a transformer block to extract information from ECG signals through two steps: the first step is learning the details of waveform variation from every lead of ECG by the CNN backbone with depthwise separable convolutions; and the second step involves combining the features extracted by CNN with positional encoding using the deformable attention transformer encoder module. Particularly, in the first step, we employ a spatial pyramid pooling layer to convert the variable dimensions of the final output feature graph into fixed-dimensional CNN features. This enables our model to accept ECG signals of varying lengths. Our CNN-DVIT network architecture exhibits exceptional performance in ECG classification on CPSC-2018 data sets, achieving an average F1 score of 82.9% across eight types of arrhythmias and sinus rhythms. These results demonstrate that deformable attention is well-suited to the unique characteristics of ECG signals and can effectively perform ECG classification tasks.

In the task of arrhythmia classification, CNN-DVIT synthesizes feature data more effectively than using any single method alone, such as LSTM, ResNet, or the transformer with the multi-head self-attention. In recent years, advancements in hardware technology, information transmission, and computing capabilities have contributed to the increasing significance of wearable ECG devices as a diagnostic modality [47]. However, our model in this study is a multi-label arrhythmia classification model for the 12-lead ECG. As such, it may not be suitable for dynamic ECG data from wearable ECG devices, which are subject to greater interference. In a clinical setting, the timeliness of the auxiliary diagnosis system is generally required. Therefore, we plan to shift our focus towards developing lightweight models with fewer parameters that are better suited for analyzing dynamic ECG data from wearables in future work.

## Figures and Tables

**Table 1:** Classification performance on CPSC-2018 data set.

Type	F1 Score						
	ResNet [30]	LSTM [8]	VGG-16 [10]	Mostayed et al., 2018 [46]	Chen et al., 2020 [22]	Che et al., 2021 [34]	CNN-DVIT
N	0.730	0.730	0.750	0.702	0.795	0.817	0.831
AF	0.882	0.792	0.861	0.815	0.897	0.858	0.924
I-AVB	0.877	0.763	0.874	0.767	0.865	0.878	0.887
LBBB	0.786	0.848	0.857	0.847	0.821	0.800	0.905
RBBB	0.905	0.909	0.918	0.898	0.911	0.872	0.935
PAC	0.487	0.268	0.333	0.397	0.734	0.618	0.704
PVC	0.733	0.763	0.859	0.807	0.852	0.830	0.842
STD	0.784	0.800	0.814	0.768	0.788	0.711	0.823
STE	0.444	0.105	0.462	0.286	0.509	0.686	0.610
Average F1	0.737	0.664	0.748	0.699	0.797	0.786	0.829

**Table 2:** Results of ablation experiments.

DMHA	MHSA	DWS-CNN	CNN	Average		
				F1	Precision	Recall
	✓	✓		0.797	0.751	0.860
	✓		✓	0.789	0.775	0.825
✓			✓	0.819	0.814	0.830
✓		✓		0.829	0.819	0.849

## Data Availability

The data presented in this study are available from the corresponding author upon reasonable request.
